# Fractionated carbon ion irradiations of the rat spinal cord: comparison of the relative biological effectiveness with predictions of the local effect model

**DOI:** 10.1186/s13014-019-1439-1

**Published:** 2020-01-03

**Authors:** Maria Saager, Christin Glowa, Peter Peschke, Stephan Brons, Rebecca Grün, Michael Scholz, Jürgen Debus, Christian P. Karger

**Affiliations:** 1 Department of Medical Physics in Radiation Oncology, German Cancer Research Center (DKFZ), Heidelberg, Germany; 2 Department of Radiation Oncology, University Hospital of Heidelberg, Heidelberg, Germany; 3 National Center for Radiation Research in Oncology (NCRO), Heidelberg Institute for Radiation Oncology (HIRO), Heidelberg, Germany; 4 Heidelberg Ion-Beam Therapy Center (HIT), Heidelberg, Germany; 5 Department of Biophysics, Helmholtz Center for Heavy Ion Research (GSI), Darmstadt, Germany; 6 Clinical Cooperation Unit Radiation Oncology, German Cancer Research Center (DKFZ), Heidelberg, Germany

**Keywords:** Carbon ion radiotherapy, Linear energy transfer (LET), Relative biological effectiveness (RBE), Dose response curves, Rat spinal cord, Local effect model (LEM)

## Abstract

**Background:**

To determine the relative biological effectiveness (RBE) and *α*/*β*-values after fractionated carbon ion irradiations of the rat spinal cord with varying linear energy transfer (LET) to benchmark RBE-model calculations.

**Material and methods:**

The rat spinal cord was irradiated with 6 fractions of carbon ions at 6 positions within a 6 cm spread-out Bragg-peak (SOBP, LET: 16–99 keV/μm). TD_50_-values (dose at 50% complication probability) were determined from dose-response curves for the endpoint radiation induced myelopathy (paresis grade II) within 300 days after irradiation. Based on TD_50_-values of 15 MV photons, RBE-values were calculated and adding previously published data, the LET and fractional dose-dependence of the RBE was used to benchmark the local effect model (LEM I and IV).

**Results:**

At six fractions, TD_50_-values decreased from 39.1 ± 0.4 Gy at 16 keV/μm to 17.5 ± 0.3 Gy at 99 keV/μm and the RBE increased accordingly from 1.46 ± 0.05 to 3.26 ± 0.13. Experimental *α*/*β*-ratios ranged from 6.9 ± 1.1 Gy to 44.3 ± 7.2 Gy and increased strongly with LET. Including all available data, comparison with model-predictions revealed that (i) LEM IV agrees better in the SOBP, while LEM I fits better in the entrance region, (ii) LEM IV describes the slope of the RBE within the SOBP better than LEM I, and (iii) in contrast to the strong LET-dependence, the RBE-deviations depend only weakly on fractionation within the measured range.

**Conclusions:**

This study extends the available RBE data base to significantly lower fractional doses and performes detailed tests of the RBE-models LEM I and IV. In this comparison, LEM IV agrees better with the experimental data in the SOBP than LEM I. While this could support a model replacement in treatment planning, careful dosimetric analysis is required for the individual patient to evaluate potential clinical consequences.

## Background

Ion beams exhibit finite ranges in tissue and allow for highly conformal irradiation of tumors by using spread-out Bragg-peaks (SOBP) [1]. Carbon ions show a significantly higher biological effectiveness than protons [2] and clinical trials are ongoing to test whether this feature improves outcome in patients [3]. The increased effectiveness of ions is measured by the *relative biological effectiveness* (RBE) given as the ratio of photon and ion doses that lead to the same biological endpoint. The RBE of carbon ions is a complex quantity and depends strongly on *linear energy transfer* (LET), fractional dose as well as on biological factors like repair capacity and others [2].

Based on early experience at the Lawrence Berkley Laboratory (USA) [4], carbon ions have been introduced clinically in 1994 at the National Institute of Radiological Sciences (Japan) [5] followed by other institutions in Germany, Japan, Italy, China and Austria [3]. When treating patients, the RBE is calculated by models [6–8] and clinical results are critically affected by their accuracy [3]. Presently, the mixed beam model (MBM) [6], the local effect model (LEM) [7] and the microdosimetric kinetic model (MKM) [8] are employed in patients. While development and initial validation of these models was mainly based on in vitro data, less effort has been performed to validate them by preclinical in vivo studies, mainly because of the limited availability of in vivo RBEs, especially for late effects.

In previous studies [9, 10], the RBE of carbon ions was determined in the rat spinal cord, which has been established as a model for late normal tissue effects [11]. These measurements were performed only in the entrance region of a mono-energetic Bragg-peak and at the center of a 1 cm SOBP and allowed for initial benchmarking of the clinically applied version of the local effect model (LEM I) at very low and high LETs. Deviations found in this comparison lead to the development of the newer version LEM IV [12], however, LEM I is still used clinically up to now and it remains to be shown, whether LEM I or LEM IV describes the RBE more accurately, as detailed information on the LET- and dose-dependence is lacking.

More recently, a large series of experiments investigated the RBE of carbon ions in the rat spinal cord after single and split doses at 6 positions within a 6 cm SOBP [13–15]. The present study extends these experiments to 6 fractions allowing for the analysis of the RBE-dependence on LET at significantly lower fractional doses as well as the dose-dependence of the RBE. These data are employed to systematically test the RBE-calculation by the RBE-models LEM I and IV.

## Methods

### Animals

This study was performed with 209 young adult female Sprague Dawley rats (208 ± 12 g, Charles River, Sulzfeld, Germany). Animals were irradiated under inhalation anesthesia with a mixture of 4% Sevoflurane (Abbott, Wiesbaden, Germany) and 2 l/min oxygen using a 50 ml disposable syringe as a mask. Experiments were approved by the governmental review committee on animal care (35–9185.81/G62–08, G117/13), and animals were kept under standard conditions at the DKFZ Center for Preclinical Research.

### Experimental setup

The experimental setup was the same as in previous studies [13–15]. The spinal cord was positioned at 6 different depths of a 6 cm SOBP (70 to 130 mm water-equivalent depth, 187–260 MeV/u), which was optimized to a uniform absorbed dose in the Bragg-peak region using the treatment planning system TRiP (treatment planning for particles) [16]. The different depths correspond to different dose-averaged LET-values (Table 1) and accordingly to different RBE-values. The field size was 10 × 15 mm^2^ and included the cervical segments C1-C6 [13–15]. The depth of the spinal cord in the SOBP was adjusted with polymethyl-methacrylate (PMMA)-boli.

**Table 1:** Dose levels and animal numbers used for the experiments

Water-equivalent depth [mm]	LET [keV/μm]	Dose levels [Gy]	Total number of animals
35	16	37^c^, 38, 39, 40, 41, 45, 49^*b*^	37
65	21	30, 32, 34, 36, 38, 40	30
80	36	25.5, 28.5^d^, 31.5, 34.5	20
100^a^	45	21.67, 24.49, 26.38, 28.26, 30.14, 32.97	30
120	66	14.5, 15.5, 16.5^*f*^, 17.5^*e*^, 18.5, 19.5, 20.5, 21.5, 22.5, 23.5, 24.5	55
127	99	15, 16, 17, 18^*e*^, 19, 20	32
		controls	5

^a^*Experiment was performed at GSI, Darmstadt*

^*b*^*One animal died due to unknown reasons*

^c^*Two animals died during irradiation narcosis*

^d^*One animal died during irradiation narcosis*

^*e*^*One animal had to be excluded due to the development of mammary carcinomas (199 d and 200 d, respectively)*

^*f*^*Two animals had to be excluded due to the development of mammary carcinomas (158 d and 257 d, respectively)*

At each depth, the spinal cord was irradiated with 6 daily fractions (Fx) of carbon ions. Animals were irradiated with different dose levels in groups of five animals (Table 1), both selected to obtain similar statistical accuracy as in previous experiments [13–15]. Doses covered 0–100% response probability and five animals were included as sham treated controls. The mid-SOBP position was irradiated at the Helmholtz Center for Heavy Ion Research (GSI), all other experiments were performed under identical conditions at the Heidelberg Ion-Beam Therapy Center (HIT). In all experiments the active raster scanning method was employed [17]. Prescribed doses refer to the maximum dose measured with a pinpoint ionization chamber (TM31009, PTW Freiburg, Germany).

### Follow-up and biological endpoint

After irradiation, rats were monitored weekly for weight and general condition. The biological endpoint was radiation induced myelopathy (paresis grade II) within 300 days, meaning that both forelimbs show signs of paralysis [9]. Rats exhibiting this endpoint were sacrificed and scored as responder.

### Data analysis

Data analysis was performed as in previous studies [9, 10, 13–15]. For each SOBP-depth, a dose-response curve and the dose at 50% complication probability, TD_50_, was determined (Appendix 1). Using the previously measured dose-response curve for 15 MeV photons [10], the RBE was calculated as the ratio of the TD_50_-values for photons and carbon ions. Including additionally data for 1 and 2 fractions [13–15], the fractionation parameter *α*/*β* and the biologically effective dose at 50% complication probability, *BED*_50_, of the linear-quadratic (LQ) model [18] were calculated for all SOBP-depths (Appendix 2). Using previously determined *BED*_50_-values for photon irradiations [9, 10], the maximum RBE in the limit of low doses was estimated as the ratio of the *BED*_50_-values of photons and carbon ions.

### RBE calculations

The RBE-values were calculated at the 6 depths of the spinal cord within the SOBP using the clinically applied LEM I [7] as well as the newer version LEM IV [12], employing the so-called ‘full-simulation’ approach [19]. The RBE was calculated at the TD_50_-dose levels obtained for carbon ions using the standard parameters for LEM I (*α*/*β* = 2 *Gy*, *α* = 0.1 *Gy*^−1^, *D*_*t*_ = 30 *Gy*) and LEM IV (*α*/*β* = 2 *Gy*, *α* = 0.003 *Gy*^−1^, *D*_*t*_ = 22 *Gy*) [20]. Maximum RBE-values were calculated from the ratio of *α*-values of carbon ions photons.

### Statistics

Dose-response curves were adjusted using the maximum likelihood procedure of STATISTICA [21]. Incomplete follow-up of animals was considered using the method of effective sample sizes [22] that corrects the number of treated and responding animals to match actuarial response rates and their variances. Standard errors (SE) of TD_50_, RBE and *α*/*β* were calculated by error propagation considering the correlation of the underlying parameters and Fieller’s Theorem [23] was used to calculate 90% confidence limits (CL). If the SE could not be calculated by STATISTICA, it was estimated as 25% of the dose difference between the neighbouring 0 and 100% dose-response levels [13].

## Results

**Fig. 1:**
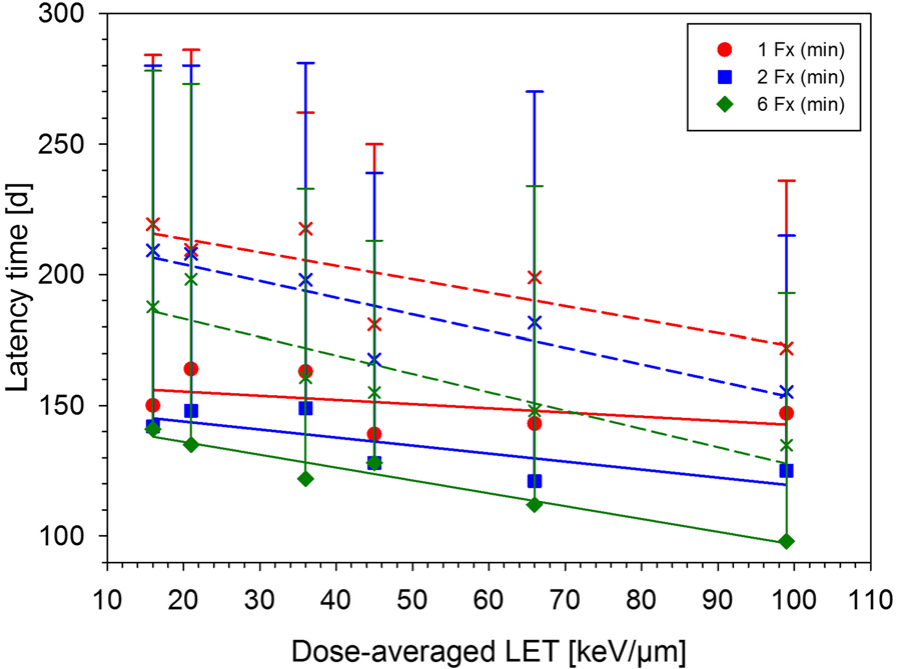
Minimum (closed symbols, solid line) and mean (crosses, dashed line) latency times for the onset of paresis grade II after carbon ion irradiation as a function of LET including data for single and split doses [13–15]. Error bars indicate the range of latency times

**Fig. 2:**
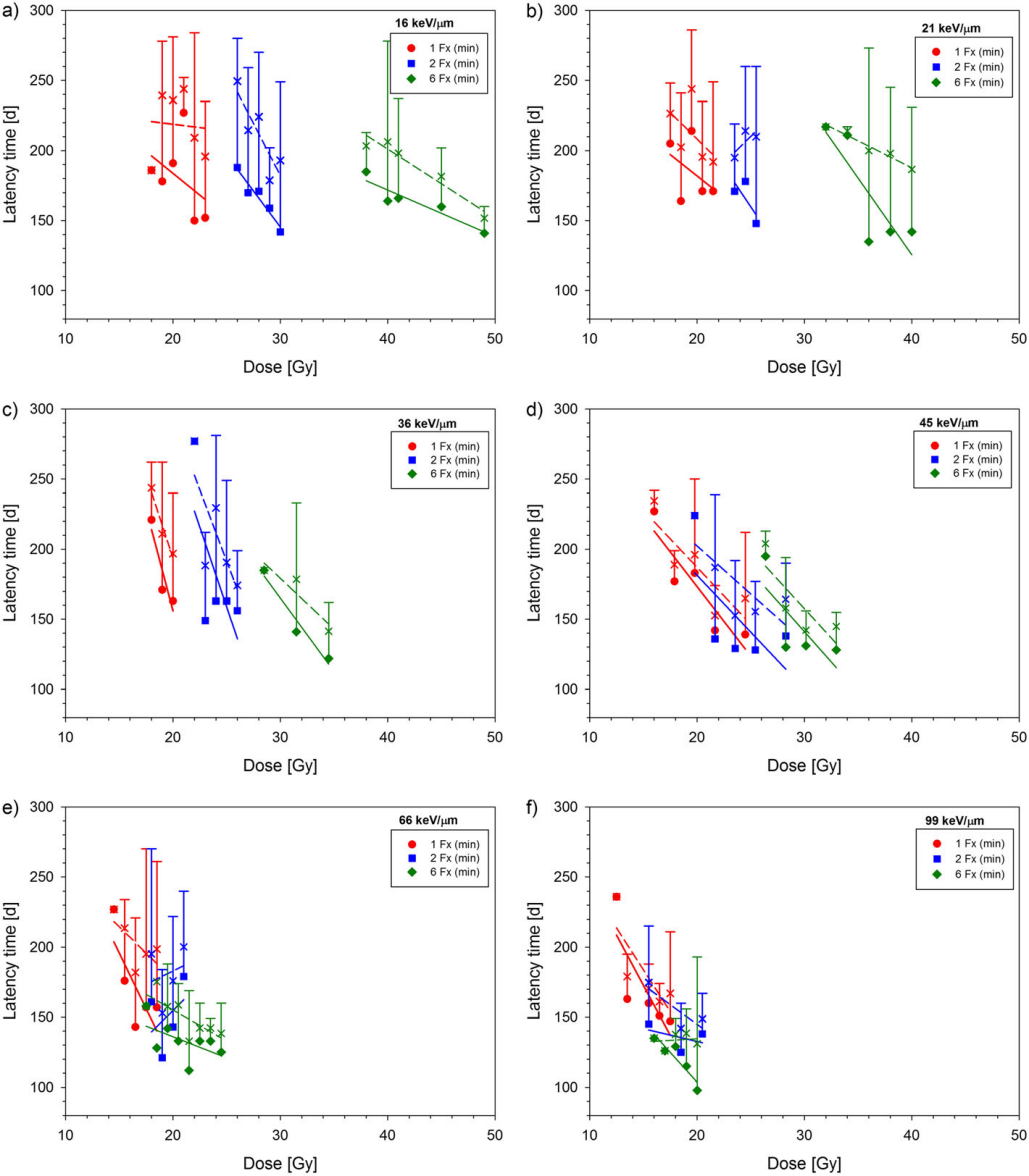
Minimum (closed symbols, solid lines) and mean (crosses, dashed lines) latency times for the onset of paresis grade II after carbon ion irradiation as a function of dose for different LETs (**a**–**f**). Data for single and split doses was obtained in previous studies [13–15]. Error bars indicate the range of latency times

Irradiation was well tolerated by the animals. Four out of 209 animals died for unknown reasons and four animals had to be excluded due to the development of mammary carcinomas (Table 1). Acute toxicity developed within 3 weeks after treatment including a slight or complete transient hair loss and moist desquamation of the skin. Mean and minimum latency time of radiation induced myelopathy decreased slightly with increasing LET, fraction number and dose (Figs. 1 and 2).

**Fig. 3:**
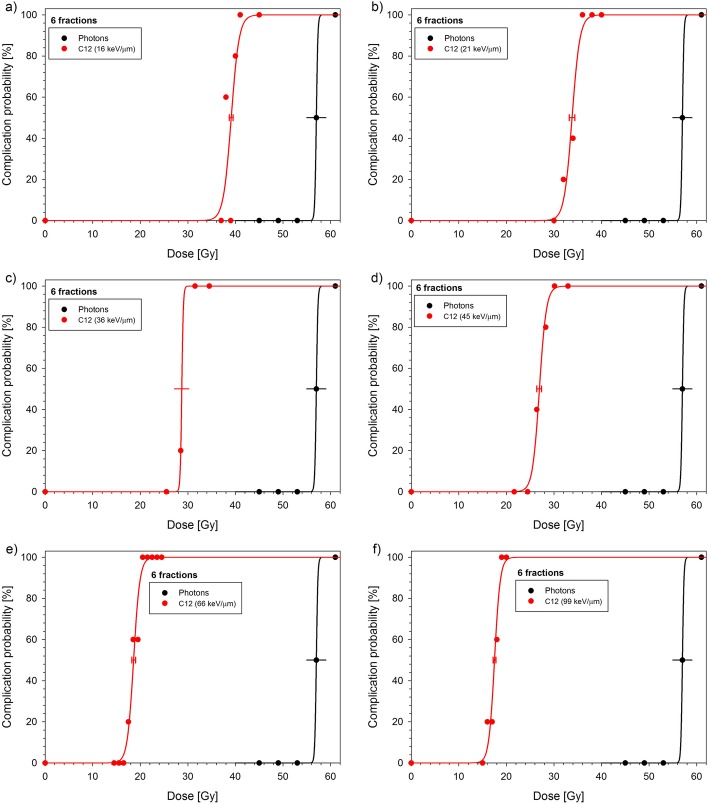
Dose-response curves after 6 Fx of carbon ions measured at different SOBP-depths (**a**–**f**) together with the previously published photon curve [10]. Error bars indicate 1 SE of TD_50_. Error bars with caps are based on the fit while those without were estimated (see text)

**Fig. 4:**
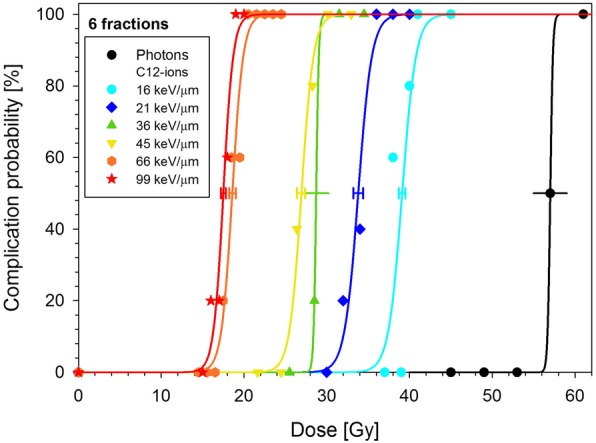
Summarized dose-response curves after 6 fractions of carbon ions as a function of LET together with the previously measured photon curve [10]. Error bars indicate 1 SE of TD_50_. Error bars with caps are based on the fit while those without were estimated (see text)

With increasing LET, the dose-response curves were shifted to lower doses (Figs. 3 and 4). This is expressed quantitatively by the TD_50_-values (Table 2A) and as a result, the RBE increased with LET. Table 2B displays the BED_50_-values and the corresponding maximum RBE-values representing the expected upper limits for very small fractional doses.

**Table 2:** Determined values for TD_50_, BED_50_, RBE, RBE_max_ and α/β together with the single standard errors (SE) and 90%-confidence limits (CL)

Study	TD_50_ ± SE (90% CL) [Gy]	RBE ± SE (90% CL)
(A) 6 Fx		
Photons^***a***^	57.0 ± 2.0 (−)^***b***^	
Carbon ions		
16 keV/μm	39.1 ± 0.4 (38.2–40.0)	1.46 ± 0.05 (1.37–1.55)
21 keV/μm	33.8 ± 0.6 (32.5–35.0)	1.69 ± 0.07 (1.58–1.80)
36 keV/μm	28.7 ± 1.5 (−)^***b***^	1.98 ± 0.12 (1.79–2.21)
45 keV/μm	26.9 ± 0.5 (25.7–28.1)	2.12 ± 0.08 (1.98–2.26)
66 keV/μm	18.6 ± 0.4 (17.8–19.3)	3.07 ± 0.12 (2.87–3.27)
99 keV/μm	17.5 ± 0.3 (16.8–18.2)	3.26 ± 0.13 (3.05–3.48)
(B) 1, 2 and 6 Fx	BED_50_ ± SE (90% CL) [Gy]	RBE_max_ ± SE (90% CL)
Photons^***a***^	244.9 ± 24.3 (208.2–293.3)	
Carbon ions		
16 keV/μm	76.3 ± 7.2 (66.0–92.0)	3.21 ± 0.44 (2.55–4.02)
21 keV/μm	53.3 ± 3.7 (47.7–60.9)	4.59 ± 0.55 (3.73–5.57)
36 keV/μm	39.5 ± 2.3 (36.0–44.6)	6.21 ± 0.71 (5.09–7.44)
45 keV/μm	36.4 ± 1.8 (33.2–40.3)	6.73 ± 0.74 (5.55–8.00)
66 keV/μm	20.3 ± 0.6 (19.4–21.3)	12.07 ± 1.24 (10.05–14.13)
99 keV/μm	19.0 ± 0.6 (18.1–20.1)	12.87 ± 1.33 (10.71–15.08)
(C) 1, 2 and 6 Fx	α/β ± SE (90% CL) [Gy]	
Photons^***a***^	2.8 ± 0.4 (2.2–3.5)	
Carbon ions		
16 keV/μm	6.9 ± 1.1 (5.2–8.9)	
21 keV/μm	9.8 ± 1.3 (7.7–12.2)	
36 keV/μm	14.6 ± 1.9 (11.4–18.2)	
45 keV/μm	12.8 ± 1.5 (10.3–16.4)	
66 keV/μm	44.3 ± 7.2 (33.8–60.4)	
99 keV/μm	30.8 ± 4.6 (23.9–40.9)	

^***a***^*Data obtained or derived from* [10]

^***b***^*Estimated standard error, confidence limits could not be determined (see text)*

**Fig. 5:**
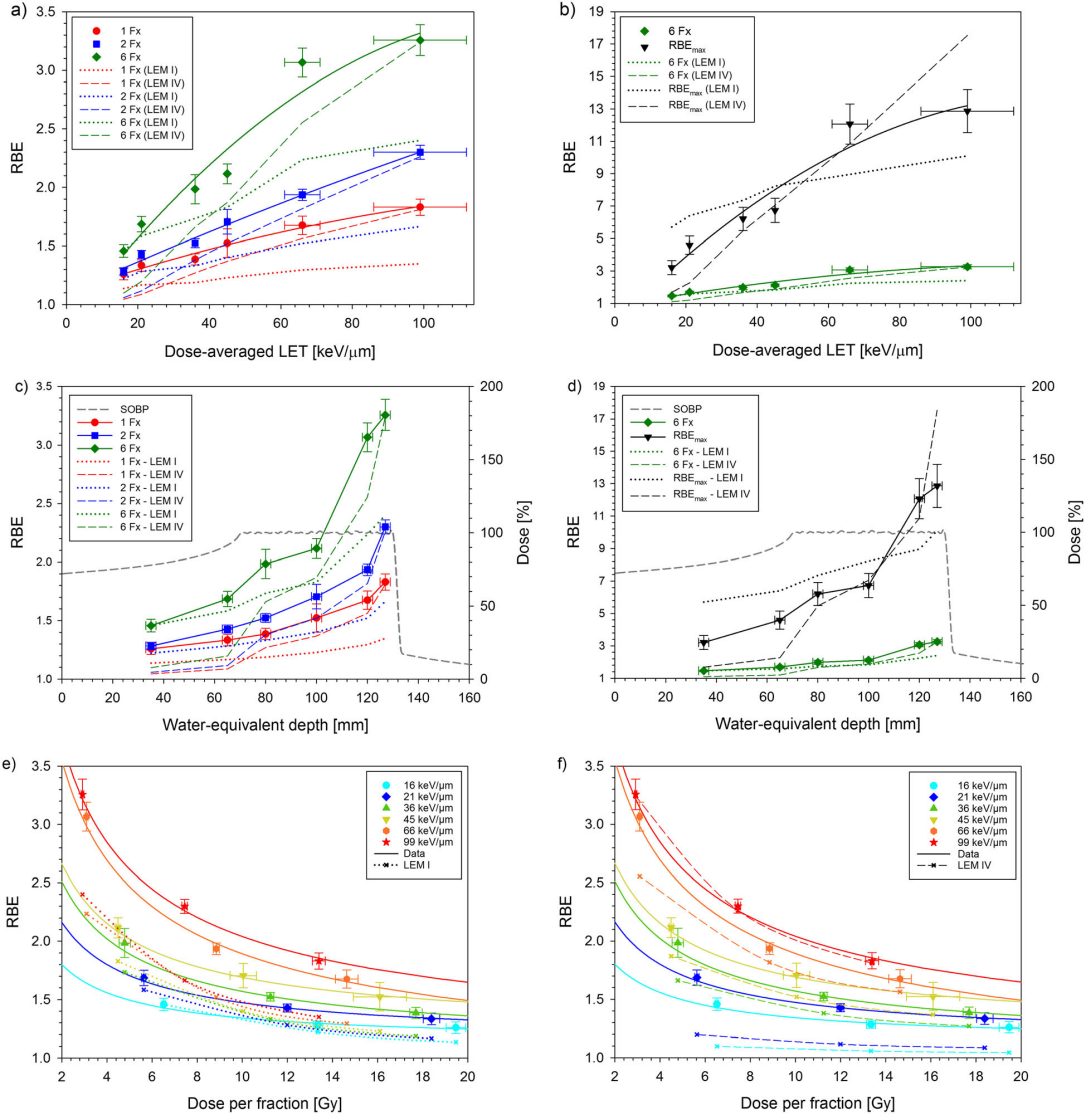
RBE as a function of LET (**a**, **b**), depth (**c**, **d**) and fractional dose (**e**, **f**) including previous data for single and split doses [13–15]. For the experimental data, the LET-dependence was fitted by 2^nd^ order polynomials while the dose-dependence was inter- and extrapolated with the LQ-model using the experimentally obtained α/β-ratios

Including previous photon data, Fig. 5 displays the dependence of the RBE and the extrapolated maximum RBE on LET, depth and fractional dose in comparison to the model predictions. Table 3 summarizes the average deviations between measured and predicted values numerically.

**Table 3:** Average deviations of LEM-predictions from experimental data. Values are given as mean ± 1 SD (A, B, D) or ± 1 SE (C), respectively

	LEM I	LEM IV
(A) Fx	RBE-deviation averaged over SOBP region^c^ [%]	
1	-20.7 ± 5.1	−6.6 ± 3.9
2	−19.9 ± 6.3	− 7.0 ± 4.1
6	−19.9 ± 7.9	−11.3 ± 7.6
∞ (limit d➔0)^a^	− 1.7 ± 25.5	4.8 ± 22.3
(B) Fx	RBE-deviation averaged over entrance region^b^ [%]	
1	−11.2 ± 1.9	−17.8 ± 1.0
2	−7.5 ± 3.8	−19.7 ± 3.0
6	−3.0 ± 4.3	−26.9 ± 3.1
∞ (limit d➔0)^a^	58.6 ± 27.2	−49.2 ± 2.2
(C) Fx	Slope ratio in SOBP region^c^	
1	0.37 ± 0.08	1.27 ± 0.21
2	0.44 ± 0.04	1.17 ± 0.08
6	0.51 ± 0.18	1.19 ± 0.35
∞ (limit d➔0)^a^	0.36 ± 0.12	1.68 ± 0.53
(D) LET [keV/μm]	RBE-deviation averaged over 1, 2 and 6 fractions [%]	
16	−4.9 ± 4.9	− 19.8 ± 4.3
21	− 9.6 ± 3.3	− 23.1 ± 5.4
36	−13.2 ± 1.0	−11.4 ± 4.3
45	− 16.9 ± 3.0	−10.9 ± 0.7
66	−23.7 ± 3.0	− 9.8 ± 6.0
99	−26.7 ± 0.8	− 1.1 ± 0.6

^a^ *Values obtained from RBE*_*max*_

^b^ *Data points at 16 and 21 keV/μm*

^c^ *Data points at 36, 45, 66 and 99 keV/μm*

While LEM I describes the RBE best at 16 keV/μm and deviates increasingly towards higher LETs, LEM IV fits best at 99 keV/μm and deviates increasingly at intermediate and low LETs (Fig. 5a). Quantitatively, LEM I differs by − 20.1% (− 19.9 – − 20.7) in the SOBP while LEM IV deviates only − 8.3%, (− 6.6 – − 11.3%) (Table 3A). In contrast, the deviations in the entrance region are larger for LEM IV (− 21.5, − 17.8% – −26.9%) than for LEM I (− 7.2, − 3.0% – −11.2%) and LEM IV generally underestimates the RBE at low LETs (Table 3B). Only the extrapolated RBE_max_-values show similar mean deviations in the SOBP for LEM I and IV (− 1.7% vs 4.8%), however they deviate increasingly but in opposite directions at low and high LETs, respectively (Fig. 5b). As compared to measurements, the slope of the LET-dependent RBE within the SOBP region was significantly more shallow for LEM I (ratio: 0.44, 0.37–0.51) and somewhat steeper for LEM IV (ratio 1.21, 1.17–1.27) (Table 3C) corresponding to a more pronounced increase of the RBE with depth (Fig. 5c,d). Finally, the experimental RBE of the single and split dose studies exhibit an essentially linear increase with LET, while the fits to the 6 fraction experiment as well as to the extrapolated maximum RBE start to saturate between 66 and 99 keV/μm.

Comparing the dose-dependence, LEM IV reproduces almost exactly the measured RBE-curve at 99 keV/μm while LEM I markedly underestimates the RBE over the whole dose range (Fig. 5e,f). At 16 keV/μm, however, the consistently increased experimental RBE of 1.3–1.5, is not described by LEM IV. This increase is better described by LEM I although the slope of the RBE with dose is larger than for the measured data. Generally, the deviations of measured and calculated RBE varied only weakly (SD of 1–6%) between the different fractionation schedules (Table 3D).

**Fig. 6:**
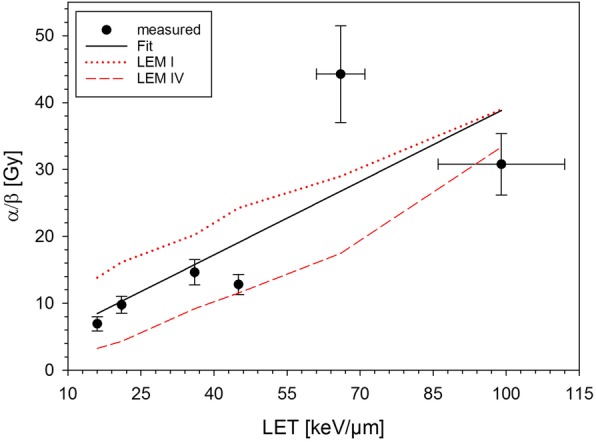
α/β-ratios at different depths within the SOBP interpolated by linear regression compared to predictions of LEM I and IV

Performing a linear regression to the experimentally obtained *α*/*β*-ratios revealed strong increase with LET (Fig. 6 and Table 2C). This is reflected by both LEM versions, however, with a systematically higher value for LEM I than for LEM IV.

## Discussion

### Methods for validating RBE-models

Clinically, the RBE-concept pursues two aims: (i) weighting the absorbed dose distribution according to the local beam quality to achieve a uniform biological effect in the SOBP, and (ii) prescribing an RBE-weighted dose being approximatively equivalent to a photon treatment. As the RBE impacts the treatment effectiveness, RBE-models require tests of increasing clinical relevance.

Initially, RBE-models were developed based on cell experiments using clonogenic survival as endpoint, thereby neglecting interactions between cells or impact of physiological and micro-environmental factors. However, especially late effects do not solely result from inactivation of cell populations [24, 25] and it is therefore important to benchmark RBE-models additionally in vivo. For this, the rat spinal cord is an established model [9–11, 26, 27] providing a well-detectable endpoint and a volume-independent response, if the irradiated segment is larger than 8 mm [28]. Hence, our study measures the RBE for the local radiation quality related to the only marginal LET-variation within the cross-section of the rat spinal cord. This is considered as the consequent next step after measuring the RBE locally for isolated cells.

We recall that the models used here predict local RBE-values at each point in the tissue and do not consider volume effects as they occur in organs of the central nervous system like e.g. the brain, where smaller irradiated volumes increase radiation tolerance and thereby reduce the risk of side effects. Disregarding the volume-effect, the rat spinal cord therefore is a particularly suitable in vivo system to benchmark the LET- and dose-dependence of RBE-models. Further developments of models and advanced experimental approaches as reported in [29] would be required to reliably disentangle high-LET-, dose- and volume-effects.

### Benchmarking LEM I and IV

The present study extends the available RBE data base to significantly lower fractional doses. Altogether, the data provides a comprehensive and consistent set of RBE-values for carbon ions as a function of LET at different dose levels. These data were used to benchmark predictions of the LEM (Fig. 5). Based on these comparisons, we conclude that (i) LEM IV agrees better in the SOBP, while LEM I fits better in the entrance region, (ii) LEM IV describes the slope of the RBE within the SOBP better than LEM I, and (iii) in contrast to the strong LET-dependence, the RBE-deviations depend only weakly on fractionation within the measured range.

Interestingly, while the RBE increased linearly with LET for single and split doses, the 6 Fx experiment as well as the extrapolated maximum RBE exhibited a slight saturation. This could be a first indication of the so-called overkill-effect, well-known from cell experiments which leads to a decreased RBE beyond 150–200 keV/μm [30].

Finally, the interpolated *α*/*β*-values rise with LET consistently with the increasingly linear cell survival curves for carbon ions. This confirms a reduced repair capacity and in spite of systematic differences between LEM I and IV, both LEM-versions describe the *α*/*β*-values reasonably well when considering experimental uncertainties.

### Clinical handling of RBE-models

Our measurements indicated that LEM IV is more accurate in the SOBP at least up to 6 fractions. Although this might suggest a replacement of LEM I in treatment planning, additional aspects need to be considered: While the RBE-weighted dose distributions optimized with both models will look very similar, the underlying RBEs will differ, leading to different absorbed doses and thus effectiveness in tumor and normal tissues. Furthermore, although the clinically applied LEM I underestimated the RBE in the rat spinal cord at high LETs, this may have been compensated in patients by prescribing a lower dose as the prescribed dose represents an independent treatment parameter. This raises the question, whether RBE-models should actually predict the absolute RBE or only its relative LET- and dose-dependence. The latter approach is followed at the Japanese centers [6, 8], where in vitro RBE-depth profiles are normalized to the clinical RBE. Moreover, the clinical RBE is not changed with fractionation and the altered effectiveness was rather considered by changing the prescribed dose [2, 3]. Thus, after successful dose finding, the remaining question is, whether a more accurate relative RBE-distribution would improve treatment outcome.

With respect to the RBE-profile, it has to be noted that the target volume contains mostly tumor rather than normal tissue. Since tumors are spatially heterogeneous, local changes of the radiation response and thus RBE are expected. As the underlying biological factors are generally not included in treatment planning, a non-uniform response within the tumor seems inevitable.

Normal tissues at risk, on the other hand, are typically located at the distal edge of the SOBP. It has been shown that optimizing the dose distribution with LEM I while assuming that LEM IV is actually correct, leads to extremely high doses in very small normal tissue volumes [20], which is in accordance with the experimental data presented here. The fact that the clinically observed incidence of normal tissue effects is nevertheless low, is thus likely attributable to a pronounced volume effect. To further tackle this aspect, a reliable volume effect model is missing. Clinically, this adds uncertainties to the expected treatment effectiveness, which may be handled by adjusting the prescribed dose while relying in the LET- and dose-dependence of the relative RBE-profile.

## Conclusion

With this study, a comprehensive in vivo data base for the RBE of carbon ions was established. This data was used to benchmark the LET- and dose-dependence of the RBE as predicted by LEM I and IV. While LEM IV agrees generally better in the SOBP, LEM I fits better in the entrance region. While this might support a model replacement in treatment planning, careful dosimetric analysis is required for the individual patient to evaluate potential clinical consequences.

## Data Availability

The datasets analyzed during the current study are available from the corresponding author on reasonable request.

## Appendix 1

For each SOBP-depth of the spinal cord, the logistic dose-response model

1a $$ P(D)=\frac{e^{b_0+{b}_1D}}{1+{e}^{b_0+{b}_1D}} $$

was adjusted using actuarial response rates. In equation 1a, *D* is the total dose and *b*_0_ and *b*_1_ are the fit-parameters, from which TD_50_, can be calculated:

1b $$ {TD}_{50}=-\frac{b_0}{b_1}. $$

## Appendix 2

For each SOBP-depth, the parameter *α*/*β* of the linear-quadratic (LQ) model [18] was estimated by adjusting the generalized logistic dose-response model

2a $$ P(D)=\frac{e^{b_0+{b}_1D+{b}_2 Dd}}{1+{e}^{b_0+{b}_1D+{b}_2 Dd}}=\frac{e^{b_0+{b}_1D\left(1+\frac{d}{b_1/{b}_2}\right)}}{1+{e}^{b_0+{b}_1D\left(1+\frac{d}{b_1/{b}_2}\right)}}=\frac{e^{b_0+{b}_1 BED}}{1+{e}^{b_0+{b}_1 BED}}, $$

to the actuarial response rates for 1, 2 and 6 fractions. In equation 2a, *D* is the total dose, *d* the fractional dose and *b*_0_, *b*_1_, *b*_2_ are fit-parameters, which allow calculating the *α*/*β*-ratio [31] by

2b $$ \frac{\alpha }{\beta }=\frac{b_1}{b_2}. $$

Using equation 2b, the expression $D\left(1+\frac{d}{b_1/{b}_2}\right)$ can be identified as the biologically effective dose, *BED* [18], and the right side of equation 2a thus describes the dose-response curve in terms of *BED* rather than absorbed dose. Accordingly, the *BED* at 50% complication probability is given by

2c $$ {BED}_{50}=-\frac{b_0}{b_1}. $$

Within the LQ-model, the *BED* describes the extrapolated isoeffective dose in the limit of zero fractional dose.

## Abbreviations

BED: Biologically equivalent dose
CL: Confidence limit
LEM: Local effect model
LET: Linear energy transfer
LQ-model: Linear-quadratic model
PMMA: Polymethyl-methacrylate
RBE: Relative biological effectiveness
SE: Standard error
SOBP: Spread-out Bragg-peak
TD: Tolerance dose
TRiP: Treatment planning for particles
